# Maternally inherited diabetes and deafness complicated by mesangial galactose-deficient IgA1 deposits: a case report

**DOI:** 10.1186/s12882-018-1152-6

**Published:** 2018-12-10

**Authors:** Keiji Sugai, Hiroyuki Ueda, Keita Morimoto, Mai Tanaka, Daisuke Takahashi, Akio Nakashima, Junichiro Kato, Hiroshi Takahashi, Yutaka Yamaguchi, Tetsuya Kawamura, Kazushige Hanaoka, Yoichi Miyazaki, Takashi Yokoo

**Affiliations:** 1 Division of Nephrology and Hypertension, Department of Internal Medicine, The Jikei University School of Medicine, Tokyo, Japan; 2 Division of Diabetes, Metabolism and Endocrinology, Department of Internal Medicine, The Jikei University School of Medicine, Tokyo, Japan; 3 Yamaguchi’s Pathology Laboratory, Chiba, Japan

**Keywords:** Maternally inherited diabetes and deafness, IgA deposits, Kidney disease, Case report, Galactose-deficient IgA1 variant

## Abstract

**Background:**

Maternally inherited diabetes and deafness (MIDD), a mitochondrial genetic disorder, typically affects the kidneys and results in end-stage renal disease. Early diagnosis of MIDD is challenging when renal manifestations precede other key clinical features such as diabetes and deafness and/or when the disease is complicated by other renal pathologies.

**Case presentation:**

Here, we present the case of a 33-year-old Japanese woman who had initially been diagnosed with IgA nephropathy but was found to have MIDD 6 years later. Two renal biopsies were conducted six years apart. While assessment of the first biopsy specimen with the monoclonal antibody (KM55) revealed mesangial IgA deposits (containing the galactose-deficient IgA1 variant [Gd-IgA1]), examination of the second specimen showed no mesangial IgA deposits and newly-developed glomerular global scleroses and tubular damage. Granular swollen epithelial cells (GSECs), characterised by abnormal mitochondria, were observed among the tubules and collecting ducts in both biopsy specimens. Mitochondrial DNA analysis revealed an m.3243A > G mutation.

**Conclusions:**

We rediscovered the usefulness of GSECs as a pathologically distinctive feature of mitochondrial nephropathy and reviewed the literature regarding MIDD complicated by mesangial IgA deposition. Furthermore, we demonstrate that the mesangial IgA deposits in this patient consisted of the galactose-deficient IgA1 variant. The monoclonal antibody (KM55) might be a useful tool to distinguish IgAN from latent IgA deposits.

## Background

Maternally inherited diabetes and deafness (MIDD) result from genetic abnormalities in mitochondrial DNA, with an A to G substitution at position 3243 (m.3243A > G) being the most common point mutation [1]. Kidneys are susceptible to mitochondrial dysfunction, and MIDD patients have a high incidence of end-stage renal disease. The prevalence of MIDD is high in Japan, and MIDD accounts for 0.9 to 5.9% of patients with diabetes who receive dialysis [2, 3].

Immunoglobulin (Ig)A nephropathy (IgAN) is the most common cause of glomerulonephritis worldwide. Previous multi-ethnic genome-wide association studies have identified risk loci resulting in a predisposition to IgAN [4–7]. Furthermore, although IgAN is prevalent worldwide, Japanese individuals carry a genetic predisposition to IgAN [6]. Therefore, nephrologists could encounter patients with MIDD, which is complicated by IgAN. However, diagnosing MIDD in patients with IgAN is challenging, particularly when renal manifestations precede diabetes or deafness. Here, we demonstrate the clinical and histological course of MIDD, complicated by IgAN, through repeated renal biopsies.

### Case presentation

A 33-year-old Japanese woman with a history of IgAN and diabetes mellitus was admitted to our hospital for the initiation of insulin therapy and evaluation of persistent proteinuria in 2015.

She had undergone a renal biopsy for proteinuria and had been diagnosed with IgAN at our hospital in 2009. At the current presentation, she had 0.7–1.0 g/day of urinary protein excretion without significant haematuria. Although her mean blood pressure was 110/60 mmHg, she was treated with an angiotensin receptor blocker (ARB) for IgAN with persistent proteinuria. Her urinary protein excretion levels had been about 0.5 g/day after the initiation of ARB. Two years later, a tonsillectomy for persistent proteinuria was performed. The patient was diagnosed with diabetes mellitus based on the fasting plasma glucose levels and haemoglobin A1c (HbA1c) levels during regular visits and was started on a dipeptidyl peptidase-4 (DPP-4) inhibitor and Pioglitazone in 2012. One year later, she discontinued both the regular visits to our hospital and her medication. Seven days prior to admission at our hospital, she visited a clinic for fatigue. Her random blood glucose level was 375 mg/dL; based on this result and persistent proteinuria, she was referred to our hospital.

**Fig. 1:**
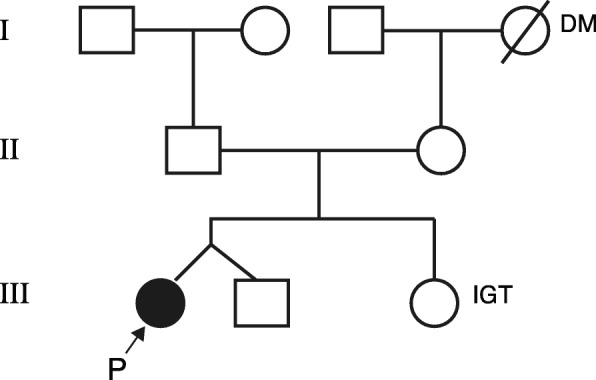
Family tree. The patient’s younger sister had impaired glucose tolerance, and her maternal grandmother was diagnosed with diabetes mellitus. DM, diabetes mellitus; IGT, impaired glucose tolerance; P, patient

Regarding her family history, her younger sister was diagnosed with impaired glucose tolerance, while her maternal grandmother was diagnosed with diabetes (Fig. 1). The physical examination was unremarkable; she had a height of 147.0 cm and weight of 46 kg (body mass index [BMI] 21.3). Laboratory testing revealed several abnormal values, including a random blood glucose level of 355 mg/dL, HbA1c level of 10.8%, 95 mmol/mol (reference; 4.6–6.2%, 27–44 mmol/mol), lactic acid level of 19.4 mg/dL (reference, 3.0–17.0 mg/dL), and pyruvic acid level of 1.28 mg/dL (reference, 0.30–0.94 mg/dL). Her renal function was preserved, as her creatinine level was 0.52 mg/dL (reference, 0.47–0.79 mg/dL) and her estimated glomerular filtration rate (eGFR) was 107.5 mL/min/1.73 m^2^. No antibodies to glutamic acid decarboxylase (GAD) or islet cells were detected. Urinalysis revealed 1+ protein and 4+ glucose without blood by dipstick. A 24-h urine collection test showed a creatinine clearance (CCr) of 175 mL/min, 2.08 g of protein with poor selectivity, and C-peptide immunoreactivity (CPR) of 33.3 μg (reference, 29.2–167 μg). During closer evaluation, the patient recalled having a hearing impairment during her school days. Therefore, audiometry was conducted and revealed mild bilateral sensorineural hearing loss > 4 kHz. Electrocardiography and echocardiography showed no abnormalities. No diabetic changes or macular retinal dystrophy were observed on funduscopic examinations.

**Fig. 2:**
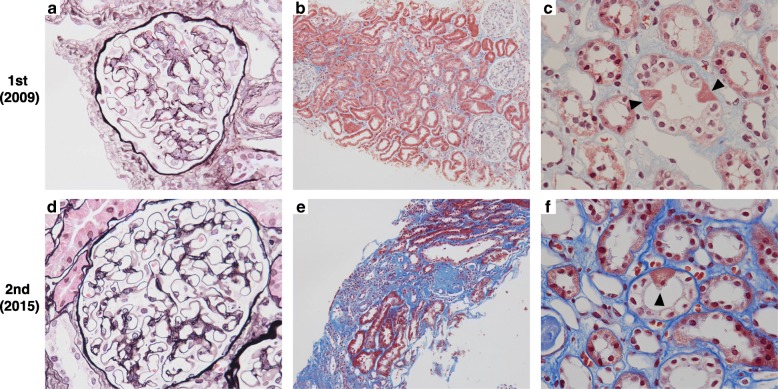
Light microscopy findings of the repeat renal biopsies. **a**-**c** show specimens from the first biopsy in 2009, and **d**–**f** depict those from the second biopsy in 2015. **a** Mesangial widening. **b** No evidence of either globally sclerosed glomeruli or tubular damage. **c** Granular swollen epithelial cells (GSECs; *arrowheads*) were widely distributed among the tubules and collecting ducts. **d** Minor glomerular abnormalities. **e** A total of 24% of glomeruli are replaced by globally sclerosed glomeruli with moderate tubular atrophy and interstitial fibrosis. **f** Abnormally distended epithelial cell (*arrowhead)* containing numerous small intracytoplasmic periodic acid-Schiff stain (PAS)-positive granules among the collecting ducts; these are identical to GSECs. **a** and **d** shown at magnification × 400, methenamine silver stain; **b** and **e** shown at magnification × 100, trichrome stain; **c** and **f** shown at magnification × 200, trichrome stain GSECs, granular swollen epithelial cells; PAS, periodic acid-Schiff stain

**Fig. 3:**
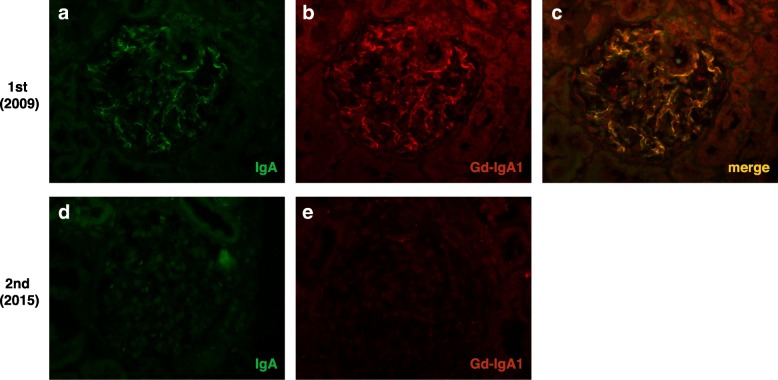
Immunofluorescence analysis of the repeat biopsy specimens. **a**–**c** are specimens from the first biopsy in 2009, whereas **d** and **e** depict those from the second biopsy in 2015. **a**–**c** Immunofluorescence using the antibody against the galactose-deficient IgA1 variant (Gd-IgA1) revealed that mesangial IgA deposits consisted of Gd-IgA1. **d**, **e** Disappearance of mesangial IgA deposits. **a**–**e** shown at magnification × 200. Gd-IgA1, galactose deficient IgA1 variant

**Fig. 4:**
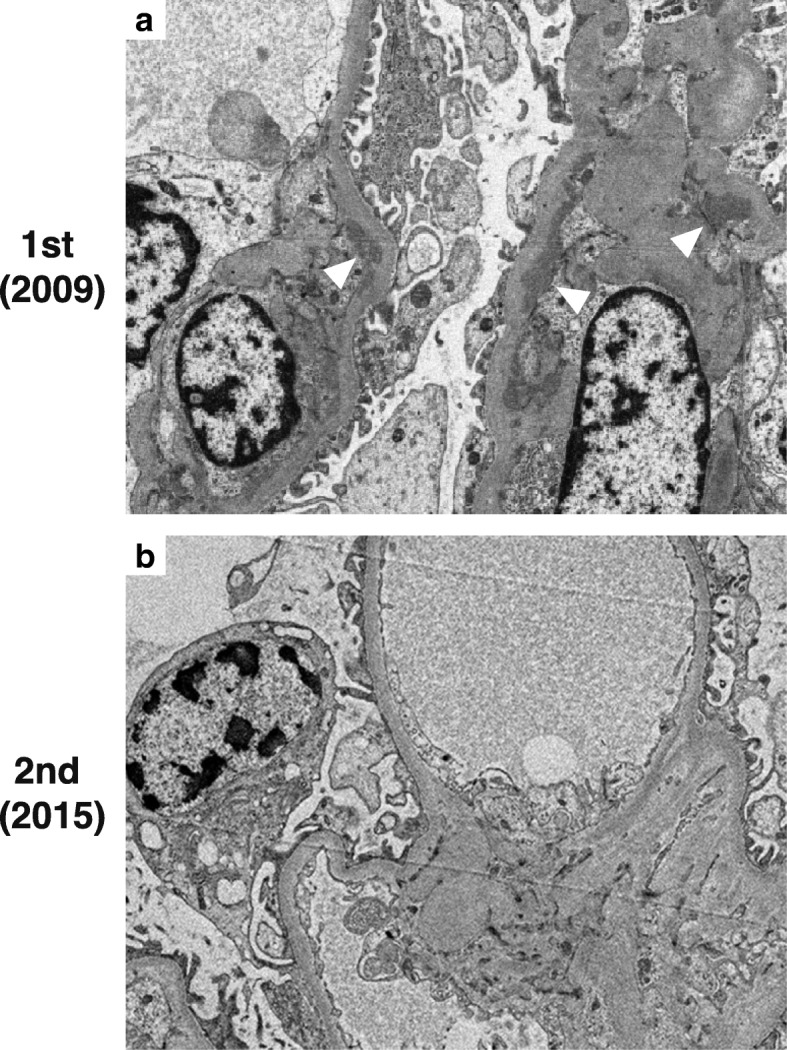
Electron microscopy of the repeat biopsy. **a** is a specimen from the first biopsy in 2009, and **b** depicts a specimen from the second biopsy in 2015. **a** Electron microscopy showing mesangial dense deposits at the first biopsy specimen (*arrowheads*). **b** Electron microscopy confirming the disappearance of mesangial IgA deposits in the second biopsy specimen. **a** and **b** shown at magnification × 6000

Based on the above findings, we suspected mitochondrial disease. A second percutaneous renal biopsy was performed to identify the cause of the persistent proteinuria in 2015 (Fig. 2d-f). The biopsy specimens for light microscopy contained 25 glomeruli, of which 6 were globally sclerotic. The remaining glomeruli revealed no mesangial hypercellularity or expansion of the matrix (Fig. 2d). There was no evidence of thickness of the glomerular basement membranes, crescents, necrotising lesions, or lesions of focal segmental sclerosis. Moderate tubular atrophy and interstitial fibrosis involving up to 30 to 40% of the sample were observed (Fig. 2e). Abnormally distended epithelial cells containing numerous small intracytoplasmic granules that were positive for periodic acid-Schiff staining were present among the collecting ducts (Fig. 2f, arrowhead). These cells were identical to ‘granular swollen epithelial cells (GSECs)’, which have previously been reported as a morphologic feature of mitochondrial nephropathy. Immunofluorescence staining for IgA and C3 was negative (Fig. 3d). On ultrastructural examination, no significant deposits were observed in the glomeruli (Fig. 4b). Podocyte foot processes were globally preserved. No cell type with an abnormal shaped or increased number of mitochondria was observed either in the glomeruli or the tubules. The GSECs that had been observed in the microscopic analysis were not apparent in the specimen for electron microscopy.

A review of the first renal biopsy specimen (Fig. 2a–c) revealed the presence of 12 glomeruli; of these, none were globally sclerosed (Fig. 2b). The glomeruli exhibited mild mesangial widening accompanied by IgA deposition (Figs. 2a, 3a, and 4a), but no crescents, mesangial hypercellularity, or segmental sclerosis. These findings correspond to M0, E0, S0, T0, and C0 in the Oxford-MEST-C classification of IgA nephropathy [8]. IgG was negative, and C3 was dimly positive on immunohistology (data not shown). We stained the first biopsy specimen with a monoclonal antibody (KM55) against Gd-IgA1 (IBL, Gunma, Japan) [9]; this immunofluorescence analysis revealed that the IgA1 deposits in the patient’s glomeruli consisted of Gd-IgA1 (Fig. 3a-c). No tubular atrophy or interstitial fibrosis were evident (Fig. 2b); however, numerous GSECs were present among the distal tubules and collecting ducts (Fig. 2c, arrowheads). On electron microscopic analysis, cells containing dysmorphic mitochondria were not apparent in the glomeruli or tubules.

Mitochondrial DNA analysis from peripheral blood revealed a m. DNA3243A > G mutation. Therefore, the patient was diagnosed with MIDD. After the initiation of insulin therapy, her blood glucose levels returned to a normal range, and she was discharged.

## Discussion

Our case demonstrates the difficulties in diagnosing mitochondrial nephropathy during the early stages of the disease, especially when it is complicated by other glomerular diseases and lacks the clinical key features such as diabetes and deafness.

An A to G substitution at position 3243 (m.3243A > G) of mitochondrial DNA affects the mitochondrial tRNA^Leu^ tertiary structure and leads to defects in the activities of complexes 1, and 4 of the respiratory chain within the mitochondria [10, 11]. Therefore, MIDD typically affects metabolically active organs such as the endocrine pancreas and cochlea, and in some cases, also the retina, muscles, kidneys, and brain. Renal manifestation sometimes precedes the diagnosis of either diabetes or deafness and can even be the sole manifestation of MIDD [12–14]. Proteinuria is a common presentation of the disease.

Focal segmental glomerular sclerotic (FSGS) lesions or tubular damage complicated by mitochondrial cytopathies are prevalent findings in the renal biopsy specimens of patients with MIDD. Electron microscopy findings of abundant and morphologically abnormal mitochondria in the cytoplasm can facilitate diagnosing mitochondrial nephropathy. However, heteroplasmy associated with mitochondrial DNA mutations often hampers the diagnosis of mitochondrial diseases through sampling errors.

The utility of GSECs for diagnosing MIDD was first described by Kobayashi et al. in 2010 who reported that GSECs were frequently observed in the collecting ducts or tubules of the kidneys of patients with MIDD [15]. The ultrastructure of GSECs is defined by abnormally shaped or increased numbers of mitochondria per cell. GSECs are observed not only in genetic mitochondrial disorders but also in miscellaneous conditions, including ageing, low birth weight, various drug toxicities, and heavy metal poisoning [16, 17]. In our case, neither FSGS lesions nor tubular damages were obvious in the first biopsy specimen. Mesangial widening and IgA deposition led us to diagnose the patient with IgAN at the time, as this also explained her persistent proteinuria. GSECs were observed in the collecting ducts in the first biopsy specimen, but the specimens for electron microscopy did not contain cells with abnormally shaped mitochondria; moreover, diabetes and deafness were not evident at the time. Six years after her initial renal biopsy, the patient underwent a second biopsy for persisting proteinuria. This second biopsy revealed a disappearance of mesangial IgA deposits with increasing numbers of global scleroses. No FSGS lesions were evident. Moderate tubular damage was observed, and there were fewer GSECs when compared to the first biopsy. The disappearance of IgA deposits in patients with IgAN has been occasionally observed after immunosuppressive treatment alone, or with tonsillectomy [18–20]. The patient in our case had undergone renin-angiotensin system (RAS) blockade therapy and tonsillectomy. Several recent studies reported that patients with IgAN had significantly higher levels of serum IgA1 levels with galactose-deficient *O*-linked glycan moieties in the hinge region (Gd-IgA1) when compared to healthy individuals. Furthermore, Gd-IgA1 has been reported to be the predominant variant of IgA1 in the mesangium of patients with IgAN [21, 22]. Using a newly developed antibody, we also confirmed that the IgA deposits in our patient contained Gd-IgA1 [9]. The origin of Gd-IgA1 has yet to be determined, but some reports suggested that the mucosal immune systems, including the tonsils, are a potential source of Gd-IgA1 [23, 24]. In this report, we could not address how serum Gd-IgA1 levels of our patient changed after tonsillectomy due to a lack of serum sample at the first biopsy. Further studies are needed to clarify whether tonsillectomy can contribute to a decrease in Gd-IgA1 production and its disappearance from the glomeruli.

In a literature search, we found four case reports (three from Japan) of mitochondrial nephropathy complicated by glomerular IgA deposition [25–28]. All patients underwent renal biopsies for persistent proteinuria before a diagnosis of mitochondrial disease was made. According to previous studies, IgA deposition was observed in 4 to 10% of consecutive necropsies without clinical evidence of renal disease [29–31] and 10 to 30% of renal allografts at transplantation [32–34]. Therefore, IgA deposition can occur in the general population without clinical signs of renal disease. All previous cases discussed the possibility of mitochondrial nephropathies being accompanied by IgA deposits. It is difficult to clearly differentiate latent IgA deposits from IgAN when the condition is complicated by other renal pathologies. Haematuria or histological findings such as mesangial cell proliferation or C3 deposition might be helpful to distinguish between the two conditions. In our case, haematuria was negative, and the pathological changes were not severe enough to explain the cause of proteinuria at the first biopsy. However, our immunofluorescence analysis using the anti-Gd-IgA1 antibody supported the possibility of an IgAN diagnosis.

## Conclusions

In this case, we illustrate the clinical and histological course of MIDD complicated by IgAN through repeated renal biopsies. Moreover, using a newly developed antibody against Gd-IgA1 revealed that the mesangial IgA deposits consisted of Gd-IgA1. This antibody might be useful to distinguish IgAN from latent IgA deposits.

## Abbreviations

ARB: Angiotensin receptor blocker
BMI: Body mass index
CC: Creatinine clearance
CPR: C-peptide immunoreactivity
DPP-4: Dipeptidyl peptidase-4
eGFR: Estimated glomerular filtration rate
FSGS: Focal segmental glomerular sclerotic
GAD: Glutamic acid decarboxylase
Gd-IgA1: galactose-deficient IgA1
GSECs: Granular swollen epithelial cells
HbA1c: Haemoglobin A1c
IgAN: Immunoglobulin (Ig)A nephropathy
MIDD: Maternally inherited diabetes and deafness
RAS: Renin-angiotensin system
